# Colonies of marine cyanobacteria *Trichodesmium* interact with associated bacteria to acquire iron from dust

**DOI:** 10.1038/s42003-019-0534-z

**Published:** 2019-08-02

**Authors:** Subhajit Basu, Martha Gledhill, Dirk de Beer, S. G. Prabhu Matondkar, Yeala Shaked

**Affiliations:** 1 The Fredy and Nadine Herrmann Institute of Earth Sciences, Hebrew University of Jerusalem, 91904 Jerusalem, Israel; 2 The Interuniversity Institute for Marine Sciences in Eilat, 88103 Eilat, Israel; 3 GEOMAR, Helmholtz Centre for Ocean Research, Wischhofstrasse 1-3, 24148 Kiel, Germany; 4 Max Planck Institute for Marine Microbiology (MPI Bremen), Celsiusstr 1, 28359 Bremen, Germany; 5 National Institute of Oceanography(CSIR), Dona-Paula, Goa, 403004 India

**Keywords:** Biogeochemistry, Ecology, Element cycles

## Abstract

Iron (Fe) bioavailability limits phytoplankton growth in vast ocean regions. Iron-rich dust uplifted from deserts is transported in the atmosphere and deposited on the ocean surface. However, this dust is a poor source of iron for most phytoplankton since dust-bound Fe is poorly soluble in seawater and dust rapidly sinks out of the photic zone. An exception is *Trichodesmium*, a globally important, N_2_ fixing, colony forming, cyanobacterium, which efficiently captures and shuffles dust to its colony core. *Trichodesmium* and bacteria that reside within its colonies carry out diverse metabolic interactions. Here we show evidence for mutualistic interactions between *Trichodesmium* and associated bacteria for utilization of iron from dust, where bacteria promote dust dissolution by producing Fe-complexing molecules (siderophores) and *Trichodesmium* provides dust and optimal physical settings for dissolution and uptake. Our results demonstrate how intricate relationships between producers and consumers can influence productivity in the nutrient starved open ocean.

## Abstract (web summary)

Subhajit Basu et al. provide evidence for mutualistic interactions between the nitrogen-fixing marine cyanobacteria *Trichodesmium* and associated bacteria in using iron from dust. Adding siderophores increases iron oxide dissolution and iron uptake, benefiting both *Trichodesmium* and the associated bacteria.

## Introduction

**Fig. 1:**
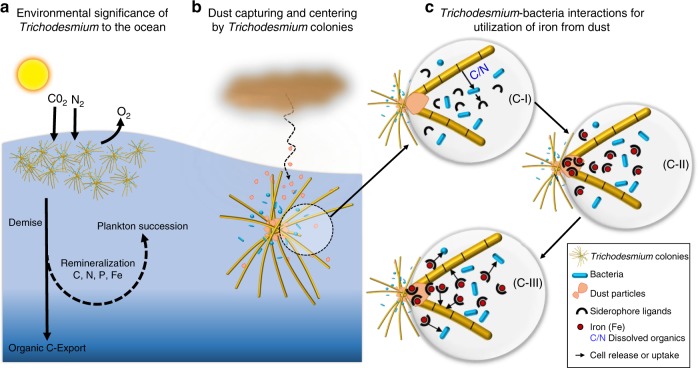
Cartoon representation of the proposed dust-bound Fe acquisition pathway employed mutually by *Trichodesmium* colonies and associated bacteria. **a** The N_2_-fixing marine cyanobacterium *Trichodesmium* spp., which commonly occurs in tropical and sub-tropical waters, is of large environmental significance in fertilizing the ocean with important nutrients. **b** *Trichodesmium* can establish massive blooms in nutrient poor ocean regions with high dust deposition, partly due to their unique ability to capture dust, center it, and subsequently dissolve it. **c** The current study explores biotic interactions within *Trichodesmium* colonies that lead to enhanced dissolution and acquisition of iron from dust. Bacteria residing within the colonies produce siderophores (c-I) that react with the dust particles in the colony core and generate dissolved Fe (c-II). This dissolved Fe, complexed by siderophores, is then acquired by both T*richodesmium* and its resident bacteria (c-III), resulting in a mutual benefit to both partners of the consortium

In large parts of the ocean, supply of the nutrients iron (Fe), phosphorous (P), and nitrogen (N) limit phytoplankton growth^1^. Some phytoplankton supply their N-demands by fixing the inert gaseous nitrogen (N_2_) into biologically accessible nitrogen and further fuel the ocean primary productivity by releasing excess fixed-nitrogen^2,3^. The cyanobacterium *Trichodesmium* spp., an important ecosystem player in oligotrophic ocean regions, contributes to ~50% of marine N_2_-fixation and forms extensive surface blooms visible even from space^4,5^ (Fig. 1a). Large fluxes of nutrients, organic molecules, and toxins released from *Trichodesmium* blooms have strong impact on both chemical and biological components of marine ecosystems^3,6,7^ (Fig. 1a). *Trichodesmium* appears typically as free filaments (trichomes) in the water-column or as colonies composed of tens to hundreds of individual trichomes^8,9^. Colonies of *Trichodesmium* host many associated bacteria which are distinct from free-living bacteria in seawater^10–12^. *Trichodesmium* and its associated bacteria exchange nutrients and organic molecules between them and act together to optimize the growth of the whole consortium^13,14^.

Atmospheric dust is considered an important source of iron to Fe-poor ocean regions, but the rapid sinking of dust from the ocean surface and the low solubility of iron from dust (dust-Fe) restricts its utilization by phytoplankton^15–17^. Buoyant *Trichodesmium* colonies overcome these constraints by efficient trapping of dust particles deposited at the ocean surface and subsequent shuffling of dust to the colony center, where it is protected from loss^18,19^ (Fig. 1b). In addition to dust capturing, *Trichodesmium* colonies were shown to chemically modify dust and increase dust-Fe solubility and bioavailability^19,20^. The two most common mechanisms microorganisms apply for dissolving mineral-Fe are reductive dissolution and siderophore promoted dissolution, both of which were suggested to play a role in *Trichodesmium*-dust interactions^19,20^. In reductive dissolution, conversion of mineral Fe(III) to soluble Fe(II) facilitates dissolution^21^. In siderophore promoted dissolution, Fe-specific ligands react with Fe(III) at the mineral surface and then the Fe-siderophore complexes return to solution^21,22^.

A large group of siderophores, produced by bacteria, fungi, and cyanobacteria, are involved in active dissolution of Fe-minerals in many terrestrial and aquatic environments^23,24^. In the ocean, siderophores from the ferrioxamine group are frequently detected in surface waters and hence are considered important for the marine Fe-cycle^24,25^. Although *Trichodesmium* captures and shuffles dust to its colony core (Fig. 1b), it does not possess known pathways for siderophore synthesis and hence in isolation cannot utilize siderophore promoted dissolution for dissolving dust-bound Fe^26^. However, some of the bacteria residing within natural *Trichodesmium* colonies have the ability to produce siderophores^26^. We therefore hypothesize that bacteria associated with *Trichodesmium* colonies increase solubility of dust-bound Fe by releasing siderophores (Fig. 1c-I) that dissolve iron from dust trapped within the colony center (Fig. 1c-II). The siderophore-mediated dust dissolution would be beneficial for *Trichodesmium* if it can utilize the Fe that is complexed by siderophores (Fig. 1c-III). In this scenario, the bacterial strategy of Fe dissolution from dust by siderophores is favorable for *Trichodesmium* and thus of mutual advantage for the consortium.

In this contribution, we explored the role of biotic interactions in actively mining dust-bound iron within *Trichodesmium* colonies. Firstly, we examined the occurance of siderophores in natural *Trichodesmium* blooms from the coastal Arabian Sea and the Gulf of Aqaba at the northern end of the Red Sea. We detected siderophores in all *Trichodesmium* blooms and observed active siderophore production in response to dust addition. Then, using radiolabeled ^55^Fe-oxyhydroxide (^55^ferrihydrite) and natural colonies from the Gulf of Aqaba, we examined the effect of siderophores on mineral-Fe dissolution and uptake by both members of the *Trichodesmium* consortium. We found that addition of siderophores increased ^55^ferrihydrite dissolution and iron uptake in natural colonies. The siderophore promoted ^55^ferrihydrite dissolution benefited both *Trichodesmium* and its associated bacteria. Lastly, using β-imaging we show that iron uptake from ^55^ferrihydrite occurred mainly in the colony center, due to reduced diffusive losses of siderophores and their Fe-complexes. Thus, the colonies form a symbiotic community where *Trichodesmium* is a primary producer, from which the colony is constructed, thereby creating a shielded microenvironment, in which bacteria make the limiting nutrient iron bioavailable for the host.

## Results

### Siderophore production by isolates of associated bacteria

First, we confirmed that associated bacteria from natural *Trichodesmium* colonies can produce siderophores in culture. We repeatedly plated individual *Trichodesmium* colonies from the Gulf of Aqaba on nutrient rich marine agar medium and isolated 23 bacterial strains. When grown in Fe-limited liquid media, the majority of these isolates (~75%) were screened as Chrome Azurol-S assay positive, which is indicative of siderophore production (Supplementary Fig. 1). These findings add up to previous genetic and physiological reports confirming the wide occurrence of this trait within *Trichodesmium’s* consortium^26,27^. In contrast, we analyzed a supernatant of cultured Fe-limited *Trichodesmium erythraeum* (strain IMS101) using high-performance liquid chromatography electrospray ionization – mass spectrometry (HPLC-ESI-MS)^28^ and observed no known siderophores.

### Occurrence of ferrioxamine siderophores in *Trichodesmium* blooms

**Fig. 2:**
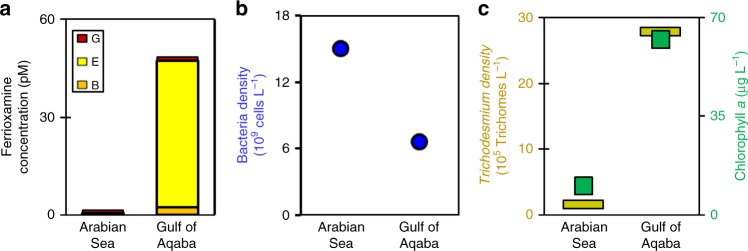
Occurrence of ferrioxamine siderophores in natural *Trichodesmium* spp. blooms from the Arabian Sea and the Gulf of Aqaba. In situ ferrioxamine (types B, E, and G) concentrations (**a**). Bacterial density in sampled blooms (**b**). *Trichodesmium* density in sampled blooms expressed as trichome counts and Chlorophyll *a* concentration (**c**)

Next, we studied the occurrence of the ubiquitous marine siderophores, ferrioxamines B, G, and E^24,25,29^ in *Trichodesmium* blooms from the Arabian Sea and Gulf of Aqaba (Supplementary Fig. 2), employing high-resolution HPLC-ESI-MS^24,28^. These surface blooms contained buoyant healthy colonies at moderate to high densities of 2–27 × 10^5^ trichomes L^−1^ (Fig. 2). Colony microbes were found at concentrations of 6–41 × 10^3^ bacteria per trichome (Supplementary Table 1), in accord with reported values from *Trichodesmium* blooms worldwide^10,30^. This density amounted to 7–15 × 10^9^ bacteria per liter (Fig. 2), which is 10–20 times higher than their density in seawater. Ferrioxamine concentrations were lower in the Arabian Sea bloom than in the Gulf of Aqaba bloom (2 pM and 45 pM, respectively, Fig. 2), and are in the range observed in surface seawater^24,25^. Normalizing in situ ferrioxamine concentrations to bacterial biomass, measured values were 0.2–3 × 10^−21^ mol per bacteria, comparable or slightly lower than ratios reported for open water in the Atlantic Ocean^24^.

### Siderophore production by *Trichodesmium* consortium in the presence of dust

**Fig. 3:**
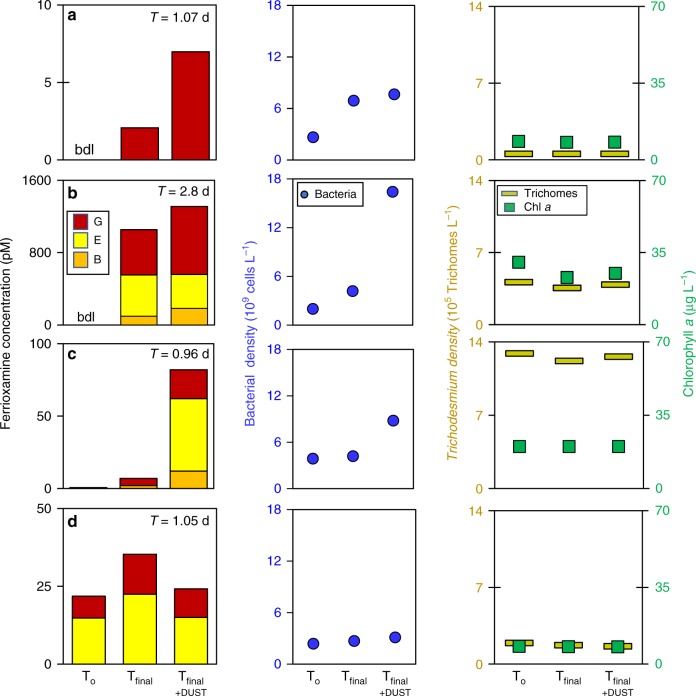
Active production of ferrioxamine siderophores from natural *Trichodesmium* blooms incubated with and without dust. Natural *Trichodesmium* blooms were carefully collected, washed, diluted, and incubated for 1–2 days with and without 2 mg L^−1^ dust in the laboratory. One experiment was conducted with a bloom from the Arabian Sea (**a**) and three separate experiments were carried out with blooms from the Gulf of Aqaba (**b**–**d**). Samples were withdrawn at the beginning (T_o_) and at the end of the incubations (T_final_, T_final+dust_). Ferrioxamine concentrations are shown on the left panels, bacteria densities on the middle panels and *Trichodesmium* biomass on the right panels. Experiment details and dates are given in Supplementary Table 1. bdl below detection limit

The presence of siderophores in *Trichodesmium* surface blooms implies that in an event of dust deposition dust-bound Fe may be dissolved by siderophores and made available to both members of the consortium. Given the high Fe requirements of *Trichodesmium* and the low solubility of Fe in dust, it is possible that the *Trichodesmium* consortium is further tuned for active mining of dust-bound Fe and may produce siderophores in respond to dust inputs. Hence, we conducted short-term incubations with natural *Trichodesmium* and probed for stimulation of ferrioxamine production by added dust. Colonies from natural blooms were washed and suspended in filtered seawater at ambient densities, while monitoring for changes in *Trichodesmium* biomass, which did not change over the course of the incubation. Bacterial cell counts, on the other hand, increased in all incubations (Fig. 3). The added local desert dust contained ~10 mg Fe g^−1^ dust, with a labile fraction of ~2 mg Fe g^−1^ dust (acetic acid extraction^31^), and it likely released additional dissolved nutrients and trace metals to the incubation water^32^.

As a result of *Trichodesmium* transfer and wash, ferrioxamine concentrations were low or below detection limit (bdl) in the initial time point of the incubations (Fig. 3, T_o_ bars). Ferrioxamine were actively produced during all incubations with final concentrations ranging from 7 pM to 1 nM, and ferrioxamine types differed among sites (Fig. 3, T_final_ bars). Dust strongly enhanced ferrioxamine production in three of the four incubations, where total ferrioxamine concentrations in the presence of dust increased by up to 12-fold compared to the incubations without dust (Fig. 3, T_final_ and T_final+Dust_ bars). Ferrioxamines were not detected in abiotic control incubations with dust only. Dust had no effect on *Trichodesmium* biomass, but it strongly enhanced bacterial growth in two of the incubations (Fig. 3). We can rule out supply of bacteria from the dust, since it was sterilized by UV irradiation prior to the experiments. Our findings on enhanced siderophore production in response to dust therefore imply that the *Trichodesmium* consortium has a mechanism to increase the supply of iron from atmospheric dust deposited in the ocean.

### Siderophores enhance Fe-mineral dissolution and bioavailability

Next, we examined the influence of ferrioxamines on dust-Fe solubility and bioavailability, using radiolabeled amorphous Fe-oxyhydroxide – ^55^ferrihydrite - as a proxy for dust. In a series of dissolution and uptake experiments, we followed the iron path from the solid to the dissolved phase and into *Trichodesmium* and its associated bacteria, implementing a method we recently optimized for this purpose^20^. We added Ferrioxamine B and E (FOB & FOE), that were detected in natural blooms (Fig. 2) to the experiments and tested their effect on ^55^ferrihydrite dissolution rates and uptake by the consortium. Furthermore, the added FOE was extracted from one of the bacteria we isolated from natural *Trichodesmium* colonies from the Gulf of Aqaba. Baseline values (controls) for non-siderophore assisted dissolution and uptake rates were obtained in the presence of heat-inactivated ferrioxamines.

**Fig. 4:**
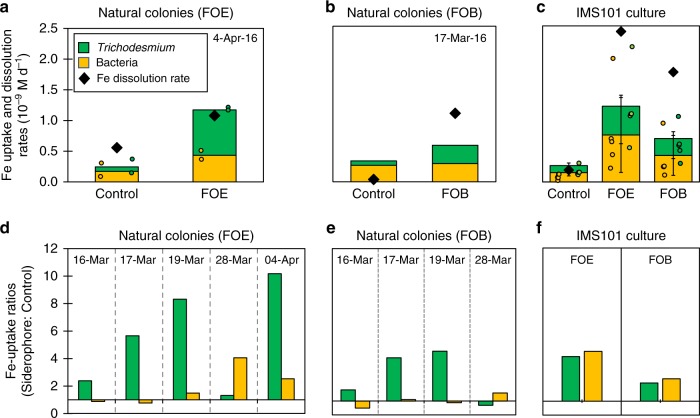
Ferrioxamines promote dissolution of ^55^ferrihydrite and enhance Fe uptake by *Trichodesmium* consortium. Top-panels **a**–**c** show experiments examining in parallel the effect of ferrioxamines B (FOB) and E (FOB) on ^55^ferrihydrite dissolution and uptake rates. ^55^Ferrihydrite dissolution rates (black diamonds) were measured in the absence of cells. ^55^Fe uptake rates from ^55^ferrihydrite were measured separately for *Trichodesmium* (green bars) and bacteria (yellow bars) and together amount for the total Fe internalized by the consortium during the experiment. Representative Fe uptake experiments of natural colonies amended with FOE (**a**) and FOB(**b**); and of cultured *T*. *Erythraeum* (IMS101) amended with both ferrioxamines (**c**).Individual data points are shown as circles. Lower panels **d**–**f** show the effect of ferrioxamines on ^55^ferrihydrite uptake, plotted as uptake rate ratios of active ferrioxamines over controls (inactivated ferrioxamines) for *Trichodesmium* (green bars) and bacteria (yellow bars). Uptake rate ratios are shown for all experiments with natural colonies amended with FOE (**d**) and FOB (**e**); and cultured *T Erythraeum* (IMS101) amended with both ferrioxamines (**f**). Error bars in **e** indicate standard deviation of replicates (*n*=5). Total uptake rates in **a** are averages of two replicates. Data are also available in Supplementary Table 2

In the absence of *Trichodesmium*, the ferrioxamines enhanced ^55^ferrihydrite dissolution rates by ~4–6-fold compared to controls with heat-inactivated siderophores (Fig. 4a–c, black diamonds), demonstrating that these compounds can dissolve Fe-minerals present in dust. Uptake experiments with natural colonies and cultures showed that the siderophore enhanced dissolution directly benefited the *Trichodesmium* consortium, with up to 10-fold higher uptake rates compared to the controls (Fig. 4). Remarkably, Fe uptake rates were of the same order of magnitude as the observed dissolution rates, which imply that all the iron that was dissolved from ferrihydrite was assimilated (Fig. 4a–c). In the cultures, both ferrioxamines had a strong and positive effect on Fe uptake (Fig. 4c), while in natural colonies FOE had a much stronger positive effect on Fe-uptake than FOB (Fig. 4a, b). These differences may reflect the larger exposure of natural colonies from the Gulf of Aqaba to FOE compared to FOB (Fig. 3b–d), and possibly hint at specificity in the uptake systems. This observation is supported by studies showing that *Trichodesmium* indeed has proteins capable of siderophore transport^33^. Interestingly, siderophore additions enhanced Fe-uptake in natural *Trichodesmium* to a greater extent than for associated bacteria (Fig. 4d–f). The lack of benefit for the bacteria from the added siderophores may indicate that these siderophore producers are sufficiently supplied with Fe even without the additions. Yet their siderophore production is also favorable for themselves, as enhanced *Trichodesmium* growth favors associated bacteria via production of exudates^2,3^.

### The confined colony core is favorable for mineral-Fe uptake

**Fig. 5:**
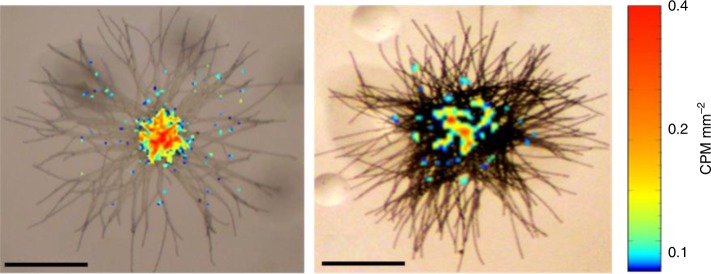
Localized Fe uptake within the core of individual *Trichodesmium* colonies incubated with ^55^ferrihydrite. Overlay of microscopic images and ^55^Fe radio-images of natural *Trichodesmium* colonies that were incubated for 24 h with ^55^ferrihydrite. The images show that only the cells in the colony core internalized ^55^Fe over this time (and hence are colored), hinting at the importance of the confined microenvironment is assisting ^55^ferrihydrite dissolution and uptake. The extracellular adsorbed ^55^Fe was removed by a Ti-EDTA-Citrate wash. Scale bar = 500 µm

Particles concentrated in the colony core provide a localized source of mineral-Fe that can be dissolved by siderophores. Hence, *Trichodesmium* and bacteria cells in the colony core may benefit from proximity to dissolved Fe and can internalize more Fe than cells in the colony periphery. We tested the spatial distribution of Fe internalized by several natural *Trichodesmium* colonies that were incubated for 24 h with 100 nM ^55^ferrihydrite, using radio imaging. Indeed, internalized Fe was detected mostly in the colony core and less in the periphery (Fig. 5). This finding of mineral-Fe utilization in the colony core further demonstrated how this consortium overcomes various physical and chemical constrains related to mineral-Fe availability. *Trichodesmium* combines physiological and behavioral traits enabling it to encounter, capture, and center dust within a microenvironment, where, assisted by its colony microbes, it dissolves mineral-Fe and effectively acquires it before it is lost by diffusion.

## Discussion

*Trichodesmium* colonies form a cohabitation that actively reacts to dust by capturing it and extracting its nutrients. The colony microenvironment is an ideal physical setting for siderophore-mediated dissolution, which is highly effective under low turbulence, high bacterial density, and short-range organism-mineral interactions. *Trichodesmium’s* ability to trap dust and confine it in the colony center, provides an optimal environment for dust dissolution by siderophores, allowing buildup of high siderophore concentrations with minimal diffusive losses of siderophores, either free or as the Fe containing complex^34^. The confined colony microenvironment is also favorable for buildup of quorum sensing molecules known to play a role in coordinating siderophore production by different bacteria^35,36^. In addition, low carbon and nitrogen resources likely limit siderophore production by free-living bacteria, while *Trichodesmium* colonies provide the substrate for bacterial colonization and large fluxes of carbon and nitrogen in the form of exudates^2,3^. Hence, bacteria residing within *Trichodesmium* colonies have a distinct advantage over free-living bacteria in dissolving and utilizing Fe-minerals. In return, *Trichodesmium* gains a source of bioavailable dissolved Fe that would have otherwise remained as insoluble dust-bound Fe. We conclude that the collaborative effort within *Trichodesmium* colonies to increase bioavailability of iron from dust is mutualistic.

Our study is unique among the many genomic-based attempts to untangle the complex *Trichodesmium*-bacteria interactions, because it provides direct experimental evidence for the actual components of dust-Fe acquisition by the consortium. We confirmed genomic predictions of siderophore production and uptake by the consortium members^26^ and support the concept that *Trichodesmium* and its colony microbes act as a synchronized metabolic circuit sharing key resources^13^. Our study adds to a growing body of research indicating that interspecies interactions control cycling of nutrients, such as nitrogen, carbon, phosphorus, iron, and vitamin B_12_^37,38^. The microbial interactions within the colonies expand *Trichodesmium’s* metabolic diversity and contribute to their success in oligotrophic systems^14,37,38^.

In the open ocean, *Trichodesmium* is often co-limited by Fe and P and relies on dust inputs to supplement the supply of these limiting nutrients^39^. Wind-driven dust deposition into the oceans is predicted to intensify due to global warming driven desertification^15^. These future climatic scenarios of increased particulate Fe (and possibly P) inputs from dust deposition are favorable for *Trichodesmium*, owing to the unique mining strategies of Fe from dust elucidated in this study. As a result, in the future ocean *Trichodesmium* may increase in abundance and by nourishing other phytoplankton with essential nutrients can accelerate ocean primary production and biogeochemical cycling of elements.

## Methods

### Collection of individual *Trichodesmium* colonies and surface blooms

Samples were obtained from two study sites: the coastal Arabian Sea)15.448°N, 73.767°E) and the Gulf of Aqaba, at the northern end of the Red Sea (29.501°N, 34.917°E). In the Arabian Sea natural *Trichodesmium* bloom was collected from surface waters with a small boat in April 2014. In the Gulf of Aqaba several transient *Trichodesmium* blooms were observed next to the pier of Interuniversity Institute for Marine Sciences during April and May 2016 (Supplementary Fig. 2). These surface blooms were collected using acid-washed wide-mouth 5L polypropylene containers, stored in Nalgene polycarbonate bottles and were immediately closed and transferred to laboratory. During spring (March-April) of 2016, individual *Trichodesmium* colonies were also collected from the Gulf of Aqaba using a static 200 µm pore-size net as described earlier^20^. In brief, *Trichodesmium* colonies were handpicked from polypropylene containers, examined for integrity under stereoscope and washed three times in chelex-cleaned filtered seawater (cFSW) prior to setting up incubation experiments. All experimental manipulations were carried out according to stringent trace metal clean protocols as further explained in the Supplementary notes.

### Characterization of ferrioxamines from natural *Trichodesmium* blooms

*Analysis of ferrioxamines*: siderophores were identified and quantified by high-performance liquid chromatography – electrospray ionization mass spectrometry (HPLC-ESI-MS; Ultimate 3000 and Q Exactive, Thermo Scientific) following preconcentration via solid-phase extraction^24,28^. Between 300 and 500 mL of incubated sample was preconcentrated over 200 mg ENV+ solid-phase extraction cartridges (Biotage) at ambient pH. Prior to analysis, columns were defrosted, washed with 10 mM ammonium carbonate (pH 8.3), and eluted with 5 mL of acetonitrile: propan-2-ol: water: formic acid (80:15:5:0.1 v:v:v:v). A 1-mL aliquot was evaporated in a centrifugal evaporator (Thermo) to a volume of ~100 µL and then diluted with 1 mL 0.1% formic acid. Weights were recorded to allow for accurate calculation of the preconcentration factor. The sample was split into four aliquots. Three aliquots were used for determination of ferrioxamine concentrations by standard addition. Standard addition was performed after addition of ferrioxamine B (Sigma), ferrioxamine G, and ferrioxamine E (EMC micro-collections, Germany). The concentrations of ferrioxamine G and E were standardized against ferrioxamine B by HPLC coupled to ICP-MS (Element XR, Thermo) after Boiteau et al.^40^. Expressed concentrations do not account for losses during preconcentration and are therefore a minimal estimate. Procedural blanks comprised of evaporated extract of ENV+ cartridges were determined to be 0.27 ± 0.11 nmol L^−1^ FOB, undetectable for FOG and 0.3 ± 0.07 for FOE (*n* = 6). One aliquot of the extract was spiked with Ga to generate Ga-siderophore complexes for isotopic mining^41^.

*Incubation set-up and filtration*: bloom samples were washed and transferred to 500 mL cFSW for incubations (Supplementary Fig. 2). Uniform distribution of colonies was achieved by preparing a stock inoculum of hand-picked colonies that was added to 1 L Nalgene bottles. Siderophores production was then examined in the incubations over 1–2 days (25 °C, 12:12 h photoperiod with ~80 µmol m^−2^ sec^−1^) in the absence and presence of dust collected at the Gulf of Aqaba (see Supplementary notes on dust collection and characterization). The dust was sterilized by UV irradiation in open petri-dishes for 30 mins to de-activate potential siderophore-producing microbes. The UV-sterilized dust was further washed in cFSW to remove potential contaminants and siderophores. The same dust was used in all incubations at a concentration of 2 mg L^−1^. Siderophores were retained on a methanol pre-activated ENV+ cartridges at the beginning and end of the incubation. Samples were pre-filtered through a 0.22-µm Acrodisc filter and loaded on ENV+ cartridges at a slow flow rate (~3 mL min^−1^) using Dynamax peristaltic pump. In situ siderophores from natural non-washed *Trichodesmium* blooms were also retained on ENV+ cartridges in a similar manner. All cartridges were stored at −20 °C prior to extraction and shipped to Germany for further processing. Biomass of *Trichodesmium* and associated bacteria were monitored in all samples - Chlorophyll a and microscopic counts of *Trichodesmium*, and DAPI counts for bacteria, as detailed further in Supplementary notes.

### Epibiont isolation, siderophore screening, and extraction

Individual *Trichodesmium* colonies were repeatedly collected from Gulf of Aqaba during winter of 2014 to isolate associated bacteria. Colonies were washed thrice with microwave sterilized filtered seawater, homogenized by vortex, plated on Zobell’s 2216E solid medium, and incubated for 72 h at 25 °C. From these plates, 23 associated bacteria colonies were isolated, purified, and screened for siderophore production, using Chrome Azurol-S assay^42^ (Supplementary Fig. 1). Siderophores were induced by growing purified isolates in Fe-limited cFSW media amended with chelex-cleaned 0.05% tryptone, phosphate (100 µM), ammonium chloride (5 µM), and MgSO_4_.7H_2_0 (50 µM), at 25 °C, shaking 150 rpm for 72 h. The cells were spun down at 10,000 rpm and 100 µL of cell-free supernatant was allowed to equilibrate with Chrome Azurol S dye in a 96-well microtiter plate (Bio-Tek) for 30 mins and absorbance measured at 630 nm. Synthetic siderophore Desferrioxamine B (1–100 µM) and blank media were used as positive and negative controls, respectively to confirm siderophore producers. The percent reduction in absorbance of Chrome Azurol S dye (630 nm) with respect to blank media was expressed as percent siderophore units.

A potent siderophore-producing epibiont strain E-23 was further grown in 1L cFSW low-Fe media and its secreted siderophores were extracted using Sep-Pak C18 columns.

In brief, stationary phase E-23 cells were spun down and the cell-free supernatant was slowly pumped through a series of three C18 Sep-Pak columns activated with methanol. The solution was circulated through the Sep-Pak three times to increase extraction yield. The columns were then eluted with three aliquots of 5 mL methanol, dried overnight in a laminar flowhood over ice, and reconstituted in 1 ml of 18.2 MΩ.cm DDW. The presence of siderophores in the C18 extract was confirmed using the Chrome Azurol-S assay. HPLC-ESI-MS analysis of this extract detected a single Fe-binding ligand, identified as ferrioxamine E (Supplementary Fig. 3). Addition of ^69^Ga showed that ferrioxamine E was the dominant siderophore, although trace amounts of ferrioxamine G were also observed. This C18 extract was not purified thereafter by preparatory chromatography, its concentration was determined using the Chrome Azurol-S assay and the extract is referred to as FOE in this publication.

### Probing the effect of siderophores on mineral Fe-uptake and dissolution rates

We used a recently optimized radiotracer assay with ^55^ferrihydrite mineral to test the effect of siderophores on ^55^ferrihydrite dissolution and uptake by natural and cultured *Trichodesmium* and bacteria, which is fully detailed in Basu and Shaked^20^.

*Mineral iron dissolution assay*: radiolabeled iron oxyhydroxide (amorphous ferrihydrite) was synthesized by titrating acidic ^55^Fe solution (^55^FeCl_3_, specific activity 10.18 mCi mg^−1^, Perkin Elmer) with 0.1 N NaOH to pH 8.1. The amorphous mineral that formed was stabilized by heating (60 °C, 2 h) and subsequent aging for 3 weeks Dissolution rates were measured in cFSW over 24 h at 25 °C in the absence of cells using 100 nM ^55^ferrihydrite, by examining the fraction smaller than 0.22 µm. Sub-samples were filtered through 0.22 µm polycarbonate filter at the beginning and end of the incubation (or in additional intermediate time points). Aliquots were placed in Quick-Safe scintillation cocktail for β-counting in Tri-carb 1600 CA (Packard) liquid scintillation counter.

*Mineral iron uptake assay*: iron internalization rates were measured by incubating either natural *Trichodesmium* (30–40 colonies per treatment) or Fe-limited cultured *Trichodesmium* erythraeum ISM101 (1.7 ± 0.22 × 10^4^ trichomes mL^−1^) with radiolabelled 100 nM ^55^ferrihydrite for 24 h (25 °C, 12:12 h photoperiod with ~80 µmol m^−2^ s^−1^)^20^. At the end of the incubation *Trichodesmium* was washed in Ti-EDTA-citrate reagent (10–20 mins) to remove all extracellular ^55^ferrihydrite. This wash not only dissolves the ^55^ferrihydrite adsorbed on the cells, but it also enables effective separation between bacteria and *Trichodesmium* due to loosening of the colony structure. We then collected *Trichodesmium* trichomes and bacteria cells separately and on 8 and 0.22 μm polycarbonates membranes, respectively. Internalized Fe by the cells on the membranes were detected using scintillation counting as above. Prior to the experiments, we examined the separation efficiency of bacteria from *Trichodesmium* and estimated that 2–5% of the bacteria that were initially present in the colonies remained with the washed *Trichodesmium*. These bacteria may account at most for 5% of the ^55^Fe uptake signal of *Trichodesmium* and hence are within the experimental error (Supplementary Table 3 and Supplementary Notes). In order to probe for the effect of siderophores on dissolution and uptake of mineral Fe, we added 1 µM of ferrioxamine B and E to the assays. Ferrioxamines B was commercially available (Sigma D9533), while ferrioxamines E was extracted from a bacterial strain E-23 isolated from natural colonies (see above). Heat-inactivated ferrioxamines were used as controls for the assays. The heat-inactivated ferrioxamines (dry heat at 120 °C for 2 h^43^) showed no Chrome Azurol-S activity.

### Radio-imaging of 2D mineral Fe uptake

Individual *Trichodesmium* colonies were incubated with 100 nM ^55^ferrihydrite in a 96-well microtiter plate for 24 h. The colonies for radio imaging were carefully picked and soaked in Ti-EDTA-Citrate solution for 10 min to remove extracellular ferrihydrite. The colonies were fixed with 2% buffered glutaraldehyde (v/v), washed by five repeated transfers in fresh FSW using droppers and placed on glass-slides. Here, by minimizing physical manipulation we managed to retain some intact colonies for the imaging. Killed individual colonies were treated as such to confirm absence of intracellular ^55^Fe. The slides were covered by a scintillation foil and the internalized ^55^Fe was radio-imaged using a Beta-Imager (Biospace Lab, Nesles la Vallée, FRANCE). Contrary to autoradiography by photographic emulsions, this method is quantitative, and imaging was terminated once 2 million counts were achieved (~3 weeks). The images were analyzed using M3Vision to determine the uptake in CPM mm^−2^. Radio-images of internalized Fe were superimposed on the colony photographs and presented as in Fig. 5 (main-text). The imaged ^55^Fe map represents uptake by *Trichodesmium* cells and not uptake by the small fraction of bacteria that escaped the separation procedure, since the amount of ^55^Fe within these bacteria is negligible (see above). Dead controls (killed with 2% glutaraldehyde for 2 h) were also imaged and revealed no measurable counts above background.

### Statistics and reproducibility

Given the low biomass of naturally occurring *Trichodesmium* population, we were unable to replicate all our measurements, let alone exceed two replicates (duplicates). However, the reproducibility of our results was attained by repeating the siderophore measurements and the uptake experiment over multiple days during two seasons, and in two remote sites. When experimenting with Fe-limited cultured *Trichodesmium erythraeum* strain IMS101, five biological replicates were conducted to ensure statistical significance.

### Reporting summary

Further information on research design is available in the Nature Research Reporting Summary linked to this article.

## Supplementary information

Reporting Summary

Supplementary Information

## Data Availability

All data supporting the findings of this study are available in the Supplementary Information file.
