# Supplementary material for: Colonies of marine cyanobacteria Trichodesmium interact with associated bacteria to acquire iron from dust
Source: Commun Biol. 2019 Aug 2;2:284. doi: 10.1038/s42003-019-0534-z (PMC6677733; doi:10.1038/s42003-019-0534-z)
Supplement: Supplementary file 1 — Reporting Summary [file 42003_2019_534_MOESM1_ESM.pdf]

nature research

Corresponding author(s): Yeala Shaked

Last updated by author(s): Jun 6, 2019

Reporting Summary

Nature Research wishes to improve the reproducibility of the work that we publish. This form provides structure for consistency and transparency in reporting. For further information on Nature Research policies, see Authors & Referees and the Editorial Policy Checklist.

Statistics

For all statistical analyses, confirm that the following items are present in the figure legend, table legend, main text, or Methods section.

n/a	Confirmed	
☐	☒	The exact sample size (n) for each experimental group/condition, given as a discrete number and unit of measurement
☐	☒	A statement on whether measurements were taken from distinct samples or whether the same sample was measured repeatedly
☒	☐	The statistical test(s) used AND whether they are one- or two-sided<br>*Only common tests should be described solely by name; describe more complex techniques in the Methods section.*
☒	☐	A description of all covariates tested
☒	☐	A description of any assumptions or corrections, such as tests of normality and adjustment for multiple comparisons
☒	☐	A full description of the statistical parameters including central tendency (e.g. means) or other basic estimates (e.g. regression coefficient) AND variation (e.g. standard deviation) or associated estimates of uncertainty (e.g. confidence intervals)
☒	☐	For null hypothesis testing, the test statistic (e.g. F, t, r) with confidence intervals, effect sizes, degrees of freedom and P value noted<br>*Give P values as exact values whenever suitable.*
☒	☐	For Bayesian analysis, information on the choice of priors and Markov chain Monte Carlo settings
☒	☐	For hierarchical and complex designs, identification of the appropriate level for tests and full reporting of outcomes
☒	☐	Estimates of effect sizes (e.g. Cohen's d, Pearson's r), indicating how they were calculated

Our web collection on statistics for biologists contains articles on many of the points above.

Software and code

Policy information about availability of computer code

Data collection	*Provide a description of all commercial, open source and custom code used to collect the data in this study, specifying the version used OR state that no software was used.*
Data analysis	MS Office 10, ChelomeEX 1.2, Image J 1.8, M3Vision, MZmine 2

For manuscripts utilizing custom algorithms or software that are central to the research but not yet described in published literature, software must be made available to editors/reviewers. We strongly encourage code deposition in a community repository (e.g. GitHub). See the Nature Research guidelines for submitting code & software for further information.

Data

Policy information about availability of data

All manuscripts must include a data availability statement. This statement should provide the following information, where applicable:

- Accession codes, unique identifiers, or web links for publicly available datasets
- A list of figures that have associated raw data
- A description of any restrictions on data availability

All data supporting the findings of this study are available in the Supplementary Information file.

Field-specific reporting

Please select the one below that is the best fit for your research. If you are not sure, read the appropriate sections before making your selection.

☐ Life sciences ☐ Behavioural & social sciences ☒ Ecological, evolutionary & environmental sciences

Ecological, evolutionary & environmental sciences study design

All studies must disclose on these points even when the disclosure is negative.

Study description	The study presents evidence for mutualistic interactions between Trichodesmium and epibiotic bacteria for dust-iron utilization, where bacteria promote dust-iron dissolution by iron-complexing molecules (siderophores) and Trichodesmium provides dust centered in its colonies and optimal physical settings for dissolution and uptake.<br>The results first demonstrate the occurrence of ferrioxamine siderophores in natural Trichodesmium sp. blooms and their active production from the Gulf of Aqaba and the Arabian Sea. Concentrations of ferrioxamines from in situ from Trichodesmium sp. blooms were quantified using high resolution LCMS-Orbitrap mass spectrometric analysis. Active production of ferrioxamines with and without dust were demonstrated in four experiments, where high-biomass incubations prepared from a stock inoculum of random hand-picked colonies from natural blooms, to ensure uniform distribution. In each experiment, the stock was split into three aliquots & diluted with 500 ml filtered seawater (Fig. 2 main text) for three different treatments. Sampling took place in the beginning of the incubation (To) prior to dust addition, and then at the end of the incubation (24-48 hr) at 25oC, for the remaining treatments (Tfinal and Tfinal+Dust). Despite large sampling efforts, the collected biomass was too low to enable duplication on a single day. Instead, the experiments were repeated on four different days (3 from Gulf of Aqaba and one from the Arabian Sea), all of which support the same findings of active siderophore production from incubated natural Trichodesmium bloom. Trichodesmium and bacteria density were monitored in each treatment at initial and final time points to examine their growth or die-off during the incubations.<br>Next, we demonstrate a positive effect of ferrioxamines on radio-labelled 55-ferrihydrite dissolution rates and Fe uptake rates by Trichodesmium-bacteria consortium and cultured Trichodesmium strain IMS101. The methodology for this Fe-uptake study was optimized as described in detail in an earlier study (Basu and Shaked 2018; See Text). Two different ferrioxamines were used in these experiments: ferrioxamine B (commercial) and ferrioxamine E. Ferrioxamine E was extracted on Sep-Pak C-18 columns from an isolated bacterial epibiont strain cultured under Fe-limitation. This strain isolated from natural 'puff' type colonies in the Gulf of Aqaba and showed highest siderophore producing potential which was identified to be exclusively as ferrioxamine E in culture media. The controls for these experiments were heat-inactivated siderophores. Natural colonies were tested in 5 different experimental days (between 16-Mar and 04-Apr 2016), three of which were replicated. Experiments with Trichodesmium culture was done with high replication(n=5).<br>Finally, radioimaging of few natural 'puff' type colonies incubated with 55ferrihydrite was done to visualize and demonstrate zone of Fe-internalization in Trichodesmium colony centers, suggesting reduced diffusive losses of siderophores and their Fe-complexes.
Research sample	1. Natural colonies (puffs and tufts) of cyanobacteria Trichodesmium spp. and surface accumulation of colonies as blooms.<br>2. Trichodesmium erythraeum IMS101 culture.
Sampling strategy	Surface static nets, Plankton Net-tows and surface collection of blooms using trace-metal clean buckets.<br>Sample Size: Sample size for natural blooms indicated in this study (Counts and Chl a) were sufficient to obtain high-resolution signals from LC-MS analysis for natural blooms. For Fe-uptake assays using radiolabelled mineral the sample size of colonies were optimized as in our earlier study (Basu and Shaked 2018; See Text)
Data collection	SB & SGPM collected the Arabian Sea samples.<br>SB, YS and MG collected the Gulf of Aqaba samples.
Timing and spatial scale	The data presented in this study were collected as follows:<br>1. Natural Trichodesmium colonies for Fe-uptake experiments in the Gulf of Aqaba from 15-Mar-2016- to 2-May-2016<br><br>2. Trichodesmium Surface bloom collections:<br>April-2014; Coastal Arabian Sea, off Goa, India.<br>20-Apr-2016 (Exp 1), 1-May-2016 (Exp 2) and 7-May-2017 (Exp 3): Gulf of Aqaba/ Eilat near InterUniversity Institute of Marine Sciences Pier, Eilat.
Data exclusions	No data is excluded.
Reproducibility	Given the low biomass of naturally occurring Trichodesmium population, we were unable to replicate all our measurements, let alone exceed two replicates (duplicates). However, the reproducibility of our results was attained by repeating the siderophore measurements and the uptake experiment over multiple days during two seasons, and in two remote sites.<br>When experimenting with Fe-limited cultured Trichodesmium erythraeum strain IMS101, five biological replicates were conduced to ensure statistical significance.
Randomization	For Fe uptake assays well-formed and integral 'puff' type colonies of different morphotypes were randomly selected and allocated for Fe-uptake assays. For high-biomass blooms, mixed colony morphotypes were randomly collected as a stock that was equally split between the different treatments and then diluted to ensure uniform distribution.
Blinding	Not Applicable.

Did the study involve field work? ☒ Yes ☐ No

Field work, collection and transport

Field conditions	Field conditions during Trichodesmium sampling (Mar-May 2016, and May 2017) can be found at Israel National Monitoring Program, Gulf of Eilat (http://www.meteo-tech.co.il/eilat-yam/eilat_instructions_en.asp).<br>Briefly, on days when ephemeral/transient surface blooms were sampled, the water-temperature varied between (23.3 - 25.1 oC), low-wind speeds of 3.1 - 2.0 m/sec, solar radiation of 858 - 955 W/m2 and a very calm sea-state.
Location	Locations:<br>1. Gulf of Aqaba - 29.5 N; 34.9 (Trichodesmium colonies - Surface - 15 m; & surface bloom)<br>2. Arabian Sea - 15.4 N, 73.7 E (Surface bloom)
Access and import/export	No permit was required for this study. AS samples were sampled by SB and SGPM. Chla and microscopic counts of Arabian Sea samples were conducted by SGPM. Frozen samples (columns with filtrates of blooms following incubations) were shipped which had no international restrictions. The sampled plankton do not fall in category of endangered species.
Disturbance	The reported sampled cyanobacteria is not an endangered species and did not cause any disturbance.

Reporting for specific materials, systems and methods

We require information from authors about some types of materials, experimental systems and methods used in many studies. Here, indicate whether each material, system or method listed is relevant to your study. If you are not sure if a list item applies to your research, read the appropriate section before selecting a response.

Materials & experimental systems

n/a	Involved in the study
☒	☐ Antibodies
☒	☐ Eukaryotic cell lines
☒	☐ Palaeontology
☒	☐ Animals and other organisms
☒	☐ Human research participants
☒	☐ Clinical data

Methods

n/a	Involved in the study
☒	☐ ChIP-seq
☒	☐ Flow cytometry
☒	☐ MRI-based neuroimaging
