# Supplementary material for: Colonies of marine cyanobacteria Trichodesmium interact with associated bacteria to acquire iron from dust
Source: Commun Biol. 2019 Aug 2;2:284. doi: 10.1038/s42003-019-0534-z (PMC6677733; doi:10.1038/s42003-019-0534-z)
Supplement: Supplementary file 2 — Supplementary Information [file 42003_2019_534_MOESM2_ESM.pdf]

Supplementary Figures

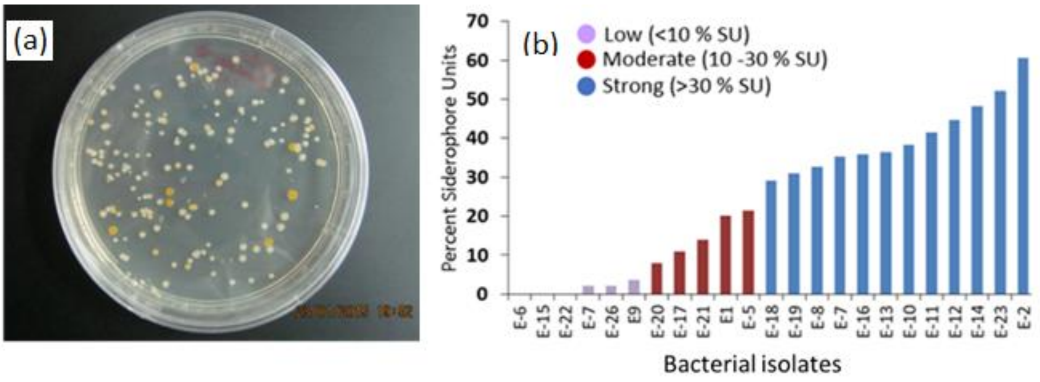

Supplementary Figure 1

(a) Typical bacteria colonies isolated from natural *Trichodesmium* colonies from Gulf of Aqaba on Zobell's 2216 E medium.

(b) Siderophore producing potential of bacterial isolates from natural *Trichodesmium* colonies. Bacteria were grown in Fe-limited cFSW media medium for 72 hrs and cell free supernatant obtained was assayed for siderophore presence using Chrome Azurol-S assay. Values are expressed as percentage reduction of the Chrome Azurol-S dye at 630 nm in sample with respect to blank-media[1].

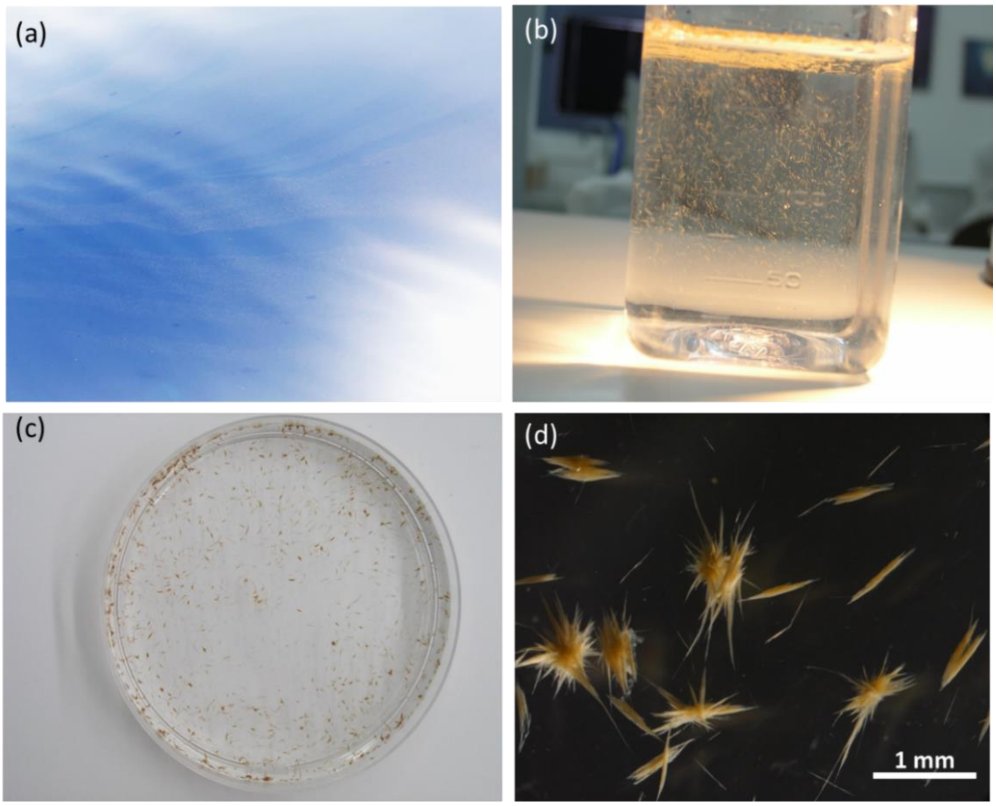

Supplementary Figure 2

Collection of *Trichodesmium* blooms for analysis of siderophores. (a) Surface bloom of *Trichodesmium* in GoA (May-2016); (b) Collected *Trichodesmium* blooms (Gulf of Aqaba, May-1-2016) resuspended for incubations in Nalgene polycarbonate bottles; (c) Colonies re-suspended in filtered seawater waiting transfer for incubations; and (d) Colony morphologies from GoA bloom.

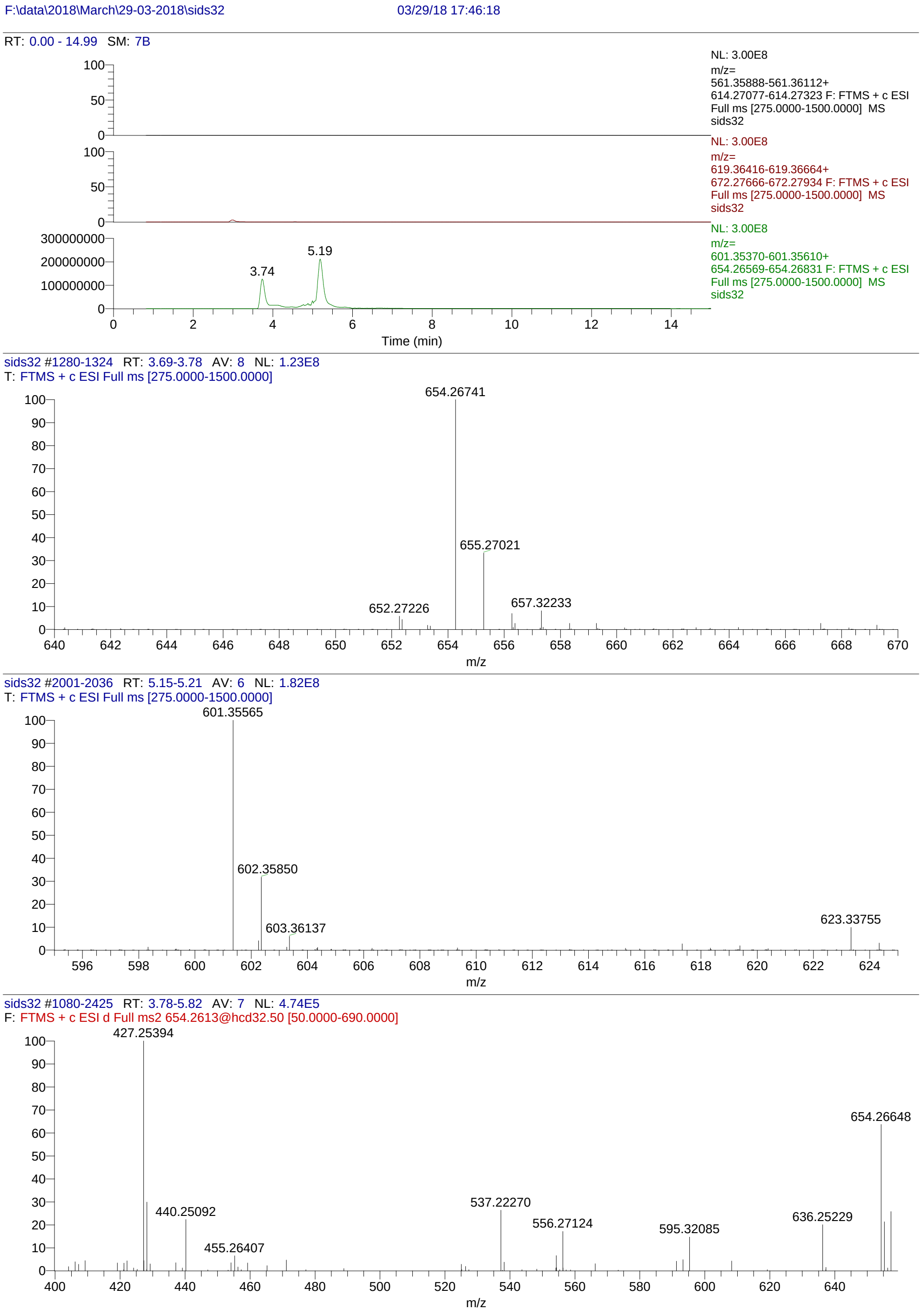

Supplementary Figure 3

HPLC-MS analysis of extract from E23 bacterial strain. Panel (A) shows extracted mass chromatograms (mass tolerance = 1 ppm) for desferrioxamine B ($[M+H]^+$ m/z 561.360, $[M-2H+Fe]^+$ m/z 614.272), desferrioxamine G ([M+H]+ m/z 619.365, $[M-2H+Fe]^+$ m/z 672.278) and desferrioxamine E ($[M+H]^+$ m/z 601.355, $[M-2H+Fe]^+$ m/z 654.267). Panels (B) and (C) shows the mass spectrum obtained for ferrioxamine E and desferrioxamine E respectively. Panel (D) shows the MS^2 spectra obtained for FOE, which resulted in product ions at m/z 636.252 and 537.223 as reported previously for FOE[2].

Supplementary Tables

Supplementary Table 1

Insitu occurrence of ferrioxamine siderophores and their concentrations during incubation assays with and without dust from Gulf of Aqaba and Arabian Sea as indicated in (Fig. 2 main text).

Area and Dates	Incubation Time (d)	Treatments	Chl*a* (µg L^{-1})	Trichodesmium Density (10^5 Trichomes L^{-1})	Bacterial Density (10^9 cells L^{-1})	Bacteria per Trichome (10^3 cells)	Bacterial Growth (µ d^{-1})	Ferrioxamines (pM)		
								B	G	E
**Arabian Sea**										
**(a) 22-Apr-14**	1.07	T_o	8.0	0.6	2.6	43		-	-	-
		T_{final}	8.04	0.5	6.9	138	0.9	-	2.1	-
		$T_{final+Dust}$	8.1	0.6	7.6	127	0.99	-	7	-
		In situ bloom	10	1.6	6.6	41		0.71	0.85	-
**Gulf of Aqaba**										
**(b) 20-Apr-16**	2.8	T_o	30	4.1	2.0	5		-	-	-
		T_{final}	22	3.6	4.2	12	0.41	96	500	457
		$T_{final+Dust}$	24	3.9	16	41	1.1	183	751	376
**(c)1-May-16**	0.96	T_o	20	13	3.9	3		0.6	-	-
		T_{final}	20	12	4.2	4	0.08	2.0	5.0	-
		$T_{final+Dust}$	20	13	8.8	7	0.82	12	20	50
		In situ bloom	62	27	15	6		2.3	1.1	45
**(d)7-May-17**	1.05	T_o	8.1	1.9	2.4	13		-	7.0	15
		T_{final}	7.9	1.7	2.7	16	0.12	-	13	22
		$T_{final+Dust}$	7.9	1.6	3.1	19	2.6	-	9.2	15
**Filtered Sea**	1.0	T_o						-	-	-
**Water**		T_{final}						-	-	-
		$T_{final+Dust}$						-	-	-

- below detection limit/not detected

Supplementary Table 2

Mineral Fe-dissolution and uptake rates from 100 nM ^{55}Ferrihydrite mineral by *Trichodesmium* and bacteria in presence and absence of siderophores. (Fig. 4 main text). Fe uptake rates from natural colonies reported as mean±SD are of two biological replicates. For cultured *Trichodesmium*, all Fe uptake rates are mean±SD for n=5.

Samples/Date	Treatments	*Trichodesmium* concentration	Fe dissolution rate (nM d^{-1})	Fe-Uptake rates (nM d^{-1})	
				Trichodesmium	**Bacteria**
**Natural Colonies**		**(Colonies mL^{-1})**			
16-Mar-2016	Control	20	0.38	0.34±0.1	1.07±0.14
	FOE		1.74	0.65±0.15	1.03±0.43
	FOB		1.65	0.49±0.04	0.58±0.09
17-Mar-2016	Control	20	0.04	0.07	0.27
	FOE		0.84	0.4	0.20
	FOB		1.1	0.29	0.29
19-Mar-2016	Control	18	0.52	0.06	0.30
	FOE		2.21	0.48	0.45
	FOB		1.93	0.27	0.27
28-Mar-2016	Control	18	0.93	0.84±0.34	0.26±0.04
	FOE		2.2	1.10±0.26	1.07±0.40
	FOB		0.95	0.58±0.05	0.42±0.04
04-Apr-2016	Control	20	0.55	0.07±0.01	0.17±0.18
	FOE		1.07	0.74±0.07	0.43±0.10
***T erythraeum* culture IMS101**		**(Trichomes mL^{-1})**			
Experiment	Control	1.2×10^4	0.19	0.11±0.04	0.15±0.05
	FOE		2.41	0.46±0.17	0.75±0.6
	FOB		1.76	0.27±0.11	0.42±0.3

Supplementary Table 3

Separation of bacteria from *Trichodesmium* in incubations. The findings from these experiments, summarized in the table below, showed that the non-washed bacteria on the 8 µm filter account for only 2.8±0.8% of total bacteria associated with the colonies. Further details of the method are provided in Supplementary notes below.

**Cold Separation**	**Total Bacteria** (10^6 cells mL^{-1})	**Bacteria in wash** (10^6 cells mL^{-1})	**Residual bacteria associated with *Trichodesmium*** (10^6 cells mL^{-1})	**Percentage of residual bacteria associated with *Trichodesmium***
Exp-1	5.38	5.08	0.11	2.0
Exp-2	7.81<br>7.50	7.20<br>7.26	0.28<br>0.20	3.6<br>2.7

Supplementary Notes

Trace Metal Clean Techniques

All experimental manipulations were carried out according to stringent trace metal clean protocols[3]. Media and culture bottles and all other plastic ware used were soaked in soap for 48 hrs, washed thoroughly in Ionized water (DI) followed by a 48 hrs wash in 10% HCl and a final rinse in double-distilled water (Milli-Q, 18.2 MΩ·cm) and stored in pre- cleaned bags. Care was taken to handle materials with trace metal clean techniques during radioactive Fe-dissolution and uptake assays from mineral ferrihydrite. Experimental manipulations were performed under positive airflow from high-efficiency particulate air (HEPA) filters, wherever deemed necessary to avoid contamination with non-radiolabelled iron. Iron was removed from the experimental medium using Chelex 100 columns. This chelex-cleaned trace metal free filtered seawater (cFSW) was used for all uptake experiments and for washing of the colonies. Growth media for cultured *Trichodesmium* (YBC-II) was prepared with double-distilled water (Milli-Q, 18.2 MΩ·cm) and analytical or higher-grade reagents in a clean-room. Iron was removed from the nutrient stocks using Chelex 100 columns and all solutions were microwave- or filter-sterilized.

Dust collection, sterilization and Fe content

Atmospheric dust transported mostly from the Sahara and the Arabian Peninsula to the Gulf of Aqaba was collected at the IUI over a period of several weeks using a home-made $1m^2$ static PVC aerosol collection device. The device was placed horizontally in a front a vertical wall to enable settling of the dust. The dust was sieved through a 200 µm mesh and stored in a desiccator. Particle size distribution was bimodal, featuring small and large particles, with an average diameter of 2.5 ± 1 µm and 21 ± 10 µm, respectively. The large particles were identified by SEM-EDS as NaCl, gypsum, feldspars, carbonates and quartz. The small particles were either in aggregates or adhered to larger particles and were too small to be analyzed individually by EDS. Soluble and acid-leached concentrations of Fe (mgFe/g dust) from this dust analyzed earlier by Torfstein et al.[4] over 24

hours varied from 0.08±0.11 in MiliQ, 2.4±1.2 in Acetic acid and 8.3±3.1 in HF-$HN0_3$ with a total Fe content of 10.7±2.5 mgFe per g dust.

***Trichodesmium* and bacterial biomass estimation**

Trichodesmium Preserved samples of *Trichodesmium* with Lugol's iodine were counted using Sedgewick-Rafter Cell (Pyser-SGI, Kent, UK) under bright-field Nikon Eclipse Ci-E microscope, 10X magnifications as described earlier[5]. For Chl *a* analysis, 2-3 ml of *Trichodesmium* samples from the incubations were filtered on 25 mm dia. GF/F filters and stored at -20°C prior to extraction with 90% acetone. Chl *a* was measured using Turner's fluorometer (Model TD700)[6]

Bacteria- Samples for bacteria were preserved with 2% glutaraldehyde (v/v). Prior to staining with DAPI (0.1 µg ml^{-1}, 15 mins), fixed-colonies were vortexed for 1 min to detach bacterial cells and obtain homogeneity. Stained samples were filtered on 0.2 µm black nuclepore filters and mounted on slides using Nikon non-fluorescent immersion oil. Cells were counted from images captured under 100X magnification using NIKON Eclipse-Ci epifluorescent microscope[7].

Separation of Bacteria from *Trichodesmium*

Prior to the uptake assays we conducted several experiments to test the separation efficiency of bacteria from natural *Trichodesmium* colonies. The separation efficiency was estimated by comparing bacterial counts in the following fractions: (1) Total bacteria, (2) Separated bacteria, and, (3) residual colony associated bacteria. The experiments included 24 hrs incubation of natural *Trichodesmium* puff-type colonies (~20 colonies mL^{-1}) with 100 nM ferrihydrite, followed by ferrihydrite removal by Ti-EDTA-Citrate wash solution, separation by size on polycarbonate membrane filters and microscopic counting of bacteria. Total bacterial counts were determined without fractionation, using Ti-EDTA-citrate washed colonies that were fixed with preservative and vortexed to dissociate bacteria. Bacteria in the

two other fractions were collected on 0.2 µm (separated bacteria) and 8 µm (residual colony associated bacteria) polycarbonate nuclepore filters. Filters were rinsed with ~3 mL of FSW five times. The filters were preserved by fixing with ~ 2 ml of 2% glutaraldehyde solution and stained with DAPI to observe bacteria at 100x magnification.

References:

1. Schwyn, B. & Neilands, H.B. Universal CAS assay for the detection and determination of siderophores. *Analytic. Biochem.* **160**, 47-56 (1987).
2. Mawji, E., Gledhill, M., Worsfold, P.J., & Achterberg, E.P. Collision-induced dissociation of three groups of hydroxamate siderophores: ferrioxamines, ferrichromes and coprogens/fusigens. *Rapid Comm. Mass. Spec.* **22**, 2195 - 2202 (2008).
3. Lis, H. & Shaked, Y. Probing the bioavailability of organically bound iron: A case study in the *Synechococcus* rich waters of the Gulf of Aqaba. *Aquat. Microb. Ecol.* **56**, 241–253 (2009).
4. Torfstein, A. et al. Chemical characterization of atmospheric dust from a weekly time series in the north Red Sea between 2006 and 2010. *Geochim. Cosmochim. Acta.* **211**, 373-393 (2017).
5. Basu, S. & Shaked, Y. Mineral iron utilization by natural and cultured *Trichodesmium* and associated bacteria. *Limnol. Oceanogr.* **63**, 2307-2320 (2018).
6. Analytical Methods of the Israel National Monitoring Program at the Gulf of Eilat. http://www.iuieilat.ac.il/Research/NMPMethods.aspx
7. Sherr, B. F., E. B. Sherr, & P. del Giorgio. in Methods in microbiology, Marine microbiology vol. 30 (ed. J. Paul) 129–159 (Academic Press, 2001).
